# Knockdown of the Autophagy Protein Beclin-1 Does Not Affect Innate Cytokine Production in Human Lung Epithelial Cells during Respiratory Syncytial Virus Infection

**DOI:** 10.3390/tropicalmed8090434

**Published:** 2023-09-04

**Authors:** Kavesha Parameswaran, Amiera Fatin Azman, Suet Lin Chia, Khatijah Yusoff, Saila Ismail

**Affiliations:** 1 Department of Microbiology, Faculty of Biotechnology and Biomolecular Sciences, Universiti Putra Malaysia, Serdang 43400, Malaysia; gs60336@student.upm.edu.my (K.P.); amieraa@sunway.edu.my (A.F.A.); suetlin@upm.edu.my (S.L.C.); kyusoff@nibm.my (K.Y.); 2 UPM-MAKNA Cancer Research Laboratory, Institute of Bioscience, Universiti Putra Malaysia, Serdang 43400, Malaysia; 3 Malaysia Genome and Vaccine Institute, National Institutes of Biotechnology Malaysia, Kajang 43000, Malaysia

**Keywords:** respiratory syncytial virus, cytokines, autophagy, airway inflammation, viral replication

## Abstract

Respiratory syncytial virus (RSV) is a major cause of respiratory tract infections in young children, globally. Autophagy is a cellular degradation process that mediates cell survival. Studies using mouse models have demonstrated that inhibiting autophagy affects the production of cytokines triggered by RSV. However, the effect of autophagy on RSV-induced cytokine production in human cells remains inadequately studied. Our previous research showed that inhibiting autophagy using pharmacological inhibitors did not affect the innate cytokine production in human lung epithelial cells (BEAS-2B) following RSV infection. In this study, we sought to validate these findings using a more specific approach, employing short-interfering RNA (siRNA) to target the important autophagy protein Beclin-1 (Bec-1). Prior to measuring cytokine production, we confirmed that silencing Bec-1 with siRNA effectively suppressed autophagy without affecting cell viability. Our results revealed that inhibiting autophagy through Bec-1 knockdown did not affect the production of innate cytokines CXCL8 and CCL5 in BEAS-2B cells during RSV infection, consistent with our previous findings using pharmacological inhibitors. Overall, our data suggest that targeting autophagy may not be an effective strategy for alleviating RSV-induced airway inflammation.

## 1. Introduction

Respiratory syncytial virus (RSV) is an enveloped, negative strand RNA virus belonging to the *Pneumoviridae* family [1]. It is an important pathogen that infects children especially in the first few months of their lives [2]. RSV infections often lead to hospitalizations as they can cause pneumonia and bronchiolitis [3,4]. Currently, RSV disease management is limited to symptomatic treatments and supportive care. Despite decades of research, it is only very recently that a vaccine against RSV was approved, and it is only for people aged 60 years or older [5]. The extent of RSV infection is established by an excessive inflammatory reaction, frequently manifested as heightened mucus production with the infiltration of neutrophils into the respiratory passages [6]. To more precisely develop therapeutic approaches capable of mitigating the intensified inflammatory response caused by RSV, it is imperative to gain a deeper understanding of the mechanisms governing cytokine production during RSV infection.

The innate immune response in the human body becomes activated when it detects viral pathogen-associated molecular patterns (PAMPs) through the pattern recognition receptors (PRRs) known as toll-like receptors (TLRs) and RIG-I-like receptors (RLRs) during RSV infection [7]. This recognition initiates a series of reactions that lead to the production and release of interferons (IFNs) such as IFN-β, as well as proinflammatory cytokines such as CXCL8. It is noteworthy that intracellular pathway autophagy has been found to play a role in TLR-mediated viral recognition by facilitating the transfer of cytosolic viral PAMPs to endosomal TLRs [8,9]. Autophagy is a conserved degradative process responsible for maintaining cellular homeostasis and assisting intracellular waste clearance including pathogen elimination. To date, numerous in vivo studies utilizing mouse models have explored the effect of autophagy inhibition on RSV-induced cytokine production [10,11,12,13,14]. However, there has been a lack of sufficient research into the effect of autophagy on cytokine production induced by RSV in human cells.

In our previous study, we showed that inhibiting autophagy with pharmacological inhibitors did not affect RSV-induced innate cytokine production in human lung epithelial cells [15], which contradicted previous publications based on mouse models. In this study, we aimed to validate these findings using a more specific approach, that is, by knocking down the expression of the autophagy protein Beclin-1 (Bec-1) using short-interfering RNA (siRNA). Bec-1 was chosen as the target gene to be knocked down due to its crucial role in the autophagy pathway [16,17].

## 2. Materials and Methods

### 2.1. Cell Culture

The BEAS-2B cell line, an immortalized human lung epithelial cell line, was obtained from the American Type Culture Collection (ATCC, Manassas, VA, USA) and cultured following established methods. In brief, the BEAS-2B cells were cultivated and maintained in Roswell Park Memorial Institute (RPMI) 1640 medium (Gibco, Billings, MT, USA) supplemented with 10% fetal bovine serum (*v*/*v*) (Gibco, USA) and incubated in a humidified incubator at 37 °C with 5% carbon dioxide (CO_2_) until they reached 80–90% confluence. Regular subculturing of the cells was performed using trypsin-EDTA (0.25%) containing phenol red (Gibco, USA).

### 2.2. Virus Propagation and Titration

The human RSV antigenic subgroup B strain 18,537 was obtained from ATCC, US. This strain was cultured in HEp-2 cervical cancer cells (HeLa contaminants; ATCC CCL-23), where cytopathic effects reached 60–70% as previously described [18]. Following this, the virus containing cell lysates were harvested through centrifugation at 1008× *g* for 7 min at 4 °C. Plaque assay using HEp2 cells was conducted to determine the viral titer as previously described [19].

### 2.3. Transient Gene Knockdown Using Short Interfering RNA (siRNA)

Beclin-1 (Bec-1) is an important autophagy protein. Bec-1 expression was silenced in BEAS-2B by short interfering RNA (siRNA) (Bioneer Corporation, Daedeok-gu, Daejeon, Republic of Korea). The target sequence of each individual siRNA used in this experiment are as previously described [20]. The siRNA was transferred into the cells using Lipofectamine™2000 (Invitrogen, Carlsbad, CA, USA); a lipid-based transfection agent. Non-targeting siRNA was used as a scrambled (scr) control. First, siRNA duplex and Lipofectamine™2000 were diluted with Opti-MEM I Reduced Serum medium at a ratio of 1:250 for a final concentration of 100 nM and 2.5:250, respectively, and incubated at room temperature for 5 min. Then, the mixtures were combined and gently vortexed for 20 min at room temperature. BEAS-2B cells were grown up to 50% confluency in a 12-well plate before siRNA transfection. The cells were then transfected with a non-targeting siRNA duplex (scr) or pooled Bec-1-targeting siRNA and mixed gently by hand-rocking and incubated for 5 h. After incubation, the medium was topped with 1 mL of fresh infection medium followed by 24 h of incubation, prior to being infected with RSV.

### 2.4. RSV Infection on BEAS-2B Cells

Following siRNA transfection (about 50–60% confluency), the BEAS-2B cells were rinsed once with 1 mL of 1X PBS and infected with RSV at MOI of 1. Cells treated with serum-free RPMI 1640 served as the RSV-uninfected control. After 2 h, the supernatant was removed, and the cells were rinsed with 1X PBS. Fresh media was added, and the cells were incubated in a humidified 37 °C incubator with 5% CO_2_ for the required time.

### 2.5. Lactate Dehydrogenase Cytotoxic Assay

To measure the percentage of cell death, a lactate dehydrogenase (LDH) assay was utilized. Prior to the assay, the cell-free supernatants and cell lysates were collected using the methods outlined in a previous study [20]. A total of 50 µL of the collected samples was added to the wells of a 96-well plate, followed by the addition of 50 µL of substrate reagent to each well. The plate was then incubated in the dark for 30 min. Finally, 50 µL of stop solution was added into each well. The absorbance was measured immediately using a microplate reader (Bio-Rad, Watford, Hertfordshire, UK) at a wavelength of 490 nm.

### 2.6. Western Blot

Preparation of protein samples was done as described in Ismail et al. (2014). Briefly, supernatant was discarded and 25 µL of lysis buffer containing protease inhibitor was added to lyse the cells. The concentration of the protein sample was determined using the Pierce bicinchoninic acid protein assay kit (Thermo Scientific, Waltham, MA, USA), following the manufacturer’s instructions. The loading solution was prepared by diluting the protein sample with a 6% sample buffer containing 2-mercaptoethanol, followed by heating the mixture in boiling water for 5 min. The final protein concentration to be subsequently added to the gel was 20 μg per well. The Bio-Rad mini PROTEIN II electrophoresis cell (Bio-Rad, UK) was used to separate proteins based on size in a 12% resolving gel and a 5% stacking gel. The gel was inserted into an electrophoresis tank filled with Tris-glycine buffer (25 mM Tris (pH 8.3), 192 mM Glycine, 1% (*w*/*v*) sodium dodecyl sulphate), before being electrophoresed at a constant current of 18 amperes per gel until all of the bromophenol blue dye had migrated to the bottom of the gel. Following electrophoresis, the separated proteins were transferred onto a polyvinylidene difluoride (PVDF) membrane using a wet transfer system. Subsequently, the PVDF membrane was then incubated with the monoclonal antibody against Bec-1 or β-actin (both from Cell Signaling Technology, Danvers, MA, USA). Afterward, the primary antibodies were removed, and the membrane was rinsed three times for 10 min each with TBST buffer (20 Mm Tris-base, 150 mM NaCl, 0.05% (*v*/*v*) Tween-20; pH 7.6). The membrane was then incubated with horseradish peroxidase (HRP)-conjugated goat anti-rabbit antibody (Cell Signaling Technology, US) for 1 h. Following the incubation, the membrane was washed three times with TBST for 10 min each and stained with WesternBright™ ECL HRP substrate (Advansta, San Jose, CA, USA) for 3 min. The blot image was captured using Amersham Imager 600 (GE Healthcare Life Sciences, Amersham, Buckinghamshire, UK).

### 2.7. Flow Cytometry

To quantify autophagy induction, Cyto-ID^®^ Autophagy Detection Kit (Enzo Life Sciences, Farmingdale, NY, USA) was utilized. Briefly, cell supernatant was removed, and the cells were washed with 1X PBS before being detached from the plate with 500 μL of trypsin-EDTA. Subsequently, the detached cells were centrifuged at 252× *g* for 5 min. The cell pellets were resuspended in 200 μL of the staining agent Cyto-ID^®^ at room temperature for 30 min in the dark. Upon completion, the stained cells were analyzed using ACEA NovoCyte Flow Cytometer (ACEA Biosciences, Santa Clara, CA, USA).

### 2.8. Cytokine Measurement by Enzyme-Linked Immunosorbent Assay (ELISA)

Cytokine concentration was measured using ELISA kits (CXCL8 kit from Invitrogen, US; and CCL5 kit from R&D System, Minneapolis, MN, USA) in a 96-well plate according to the manufacturer’s instructions. The cytokine concentrations were determined using matched antibody pairs. After incubation with the detecting antibodies, the plate was washed three times with wash buffer (1X PBS in 0.05% Tween-20) before being incubated with 100 μL of 1X 3,3′,5,5′-tetramethylbenzidine solution at room temperature for 15 min. Subsequently, 100 μL of the stop solution (1 M phosphoric acid) was added to each well. A microplate reader (Bio-Rad, UK) was used to measure the absorbance at a wavelength of 450 nm.

### 2.9. Statistical Analyses

Data are presented as mean ± standard deviation (SD). Statistical analyses were performed using GraphPad Prism v6.0 software.

## 3. Results

### 3.1. Cell Viability Is Not Affected by the Knockdown of Bec-1

**Figure 1:**
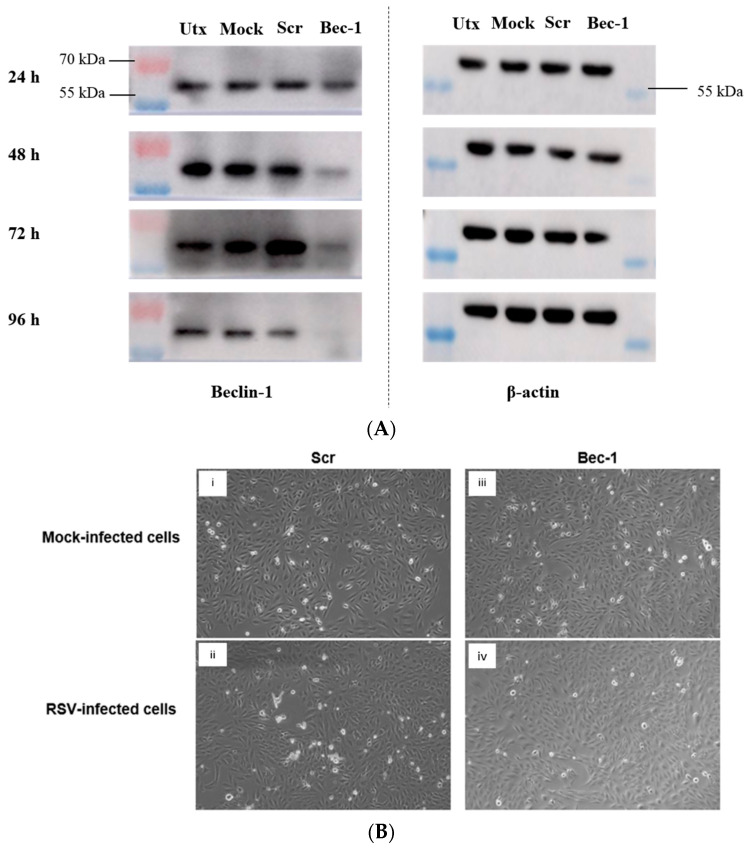

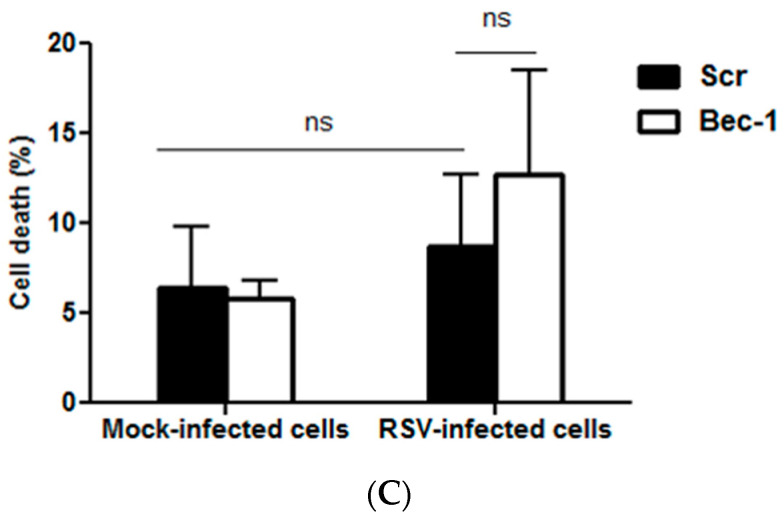
Bec-1 protein is notably reduced following siRNA transfection and the Bec-1 knockdown does not cause cell death in BEAS-2B lung epithelial cells. (**A**) Cells were either left untransfected (Utx), transfected with the transfecting reagent alone (Mock) or transfected with a non-targeting scrambled control siRNA (Scr) or siRNA targeting Bec-1 at 100 nM. Whole cell lysates were harvested at 24, 48, 72 and 96 h post-transfection. Western blot was performed to analyze Bec-1 protein expression. β-actin protein expression was also analyzed as the housekeeping protein. Data shown are representative images of *n* = 2 independent experiments. (**B**) Cells were transfected with non-targeting siRNA (Scr control) or Bec-1-targeting siRNA for 24 h before being left uninfected (mock) or infected with RSV at MOI of 1. After 48 h post-infection (h.p.i.), cell morphology was visualized using a phase-contrast microscope. (**C**) LDH assay was also carried out to quantify percentage of cell death. Data shown are mean ± SD of *n* = 3 independent experiments. ns: not significant.

Prior to investigating the effect of siRNA-mediated Bec-1 knockdown on cytokine production, we sought to confirm the successful reduction of Bec-1 protein expression following siRNA transfection. As shown in Figure 1A, transfection with siRNA targeting Bec-1 remarkably reduced Bec-1 protein expression at 24, 48, 72 and 96 h post-transfection.

We also assessed whether the siRNA-mediated knockdown of Bec-1 could affect cell viability. As depicted in Figure 1B, Bec-1 knockdown did not affect the cell morphology and viability, neither with (ii and iv) nor without (i and iii) RSV infection, as observed at 92 h post-transfection (48 h.p.i.). To further validate the results, LDH assay was performed to quantify the percentage of cell death. Similar to the microscopic observations, the percentages of cell death of the Bec-1 siRNA-transfected cells were comparable to those of the Scr siRNA control samples, similarly, both with or without RSV infection (Figure 1C).

### 3.2. Bec-1 Knockdown Inhibits RSV-Induced Autophagy in BEAS-2B Lung Epithelial Cells

**Figure 2:**
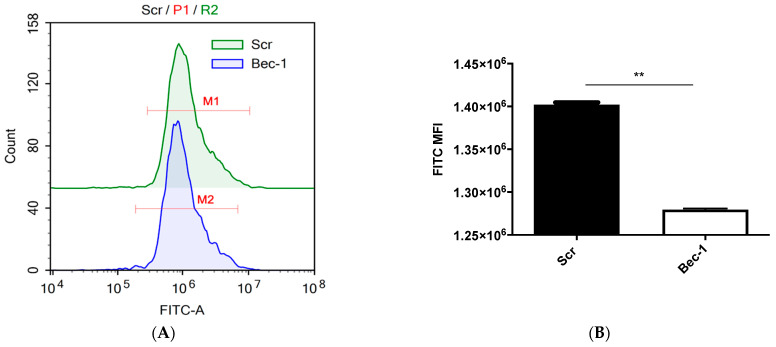
RSV-infected cells transfected with Bec-1-targeting siRNA shows a significant reduction in autophagic activity in BEAS-2B lung epithelial cells. (**A**) Cells were transfected with Scr control or Bec-1-targeting siRNA. At 24 h post-transfection, cells were infected with RSV at MOI of 1. At 24 h.p.i., cells were stained with the Cyto-ID autophagy detection kit and autophagic flux was quantified by flow cytometry. (**A**) Flow cytometry histogram profile showing autophagic flux inhibition by Bec-1-targeting siRNA (shown in blue) compared to the Scr control (shown in green). (**B**) Column graph of mean fluorescence intensity (MFI) representing the autophagic activity. MFI was obtained from the ranged gates labelled M1 (Scr) and M2 (Bec-1) shown in the histogram. Data shown are ±SD of *n* = 2 independent experiments. Significant difference is indicated by ** for *p* < 0.01, analyzed by paired *t*-test.

In our previous study [15], we demonstrated that RSV could induce autophagy in BEAS-2B cells. To confirm the effectiveness of Bec-1-targeting siRNA in suppressing RSV-induced autophagy, we utilized the CYTO-ID autophagy detection kit, which contains a green fluorescent dye that selectively labels autophagosomes. Subsequently, the green fluorescent signal was quantified by flow cytometry. Remarkably, the siRNA-mediated knockdown of Bec-1 exhibited a substantial reduction in RSV-induced autophagy, in comparison to the non-targeting scrambled (scr) control (Figure 2).

### 3.3. Bec-1 Knockdown Does Not Affect RSV-Induced Innate Cytokine Production in Lung Epithelial Cells

**Figure 3:**
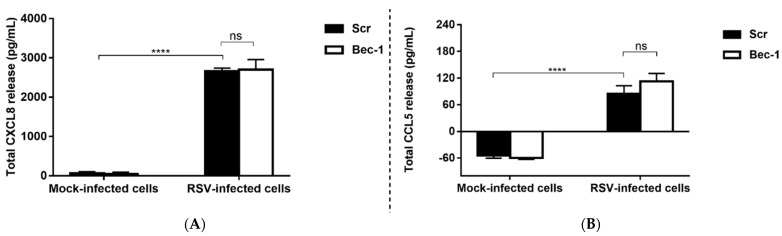
Inhibition of autophagy by Bec-1 knockdown does not affect innate cytokine production in lung epithelial cells. BEAS-2B cells were transfected with a non-targeting scrambled control siRNA (Scr) or Bec-1-targeting siRNA. After 24 h, cells were either left uninfected or infected with RSV at MOI of 1 for 48 h. The release of CXCL8 and CCL5 was measured using ELISA. Production of (**A**) CXCL8 and (**B**) CCL5 is not affected by inhibition of autophagy by Bec-1-targeting siRNA. Data shown are mean ± SD of *n* = 3 independent experiments. Significant difference is indicated by **** for *p* < 0.0001, analyzed by two-way ANOVA with Tukey’s post-test. ns: not significant; LDH: lactate dehydrogenase.

After confirming that siRNA-mediated Bec-1 knockdown does not compromise cell viability, the study proceeded to examine the effect of Bec-1 knockdown on cytokine production in RSV-infected cells. The release of chemokines C-X-C motif ligand 8 (CXCL8) and C-C motif ligand 5 (CCL5) induced by RSV was measured by ELISA. CXCL8 and CCL5 are potent neutrophil and eosinophil attractants, respectively [21]. Infiltration of neutrophils and eosinophils into the airway is strongly associated with disease severity caused by RSV infection [6]. Consistent with our previous study employing pharmacological autophagy inhibitors [15], siRNA-mediated knockdown of the autophagy protein Bec-1 did not affect the production of either innate cytokine (Figure 3).

## 4. Discussion

RSV is recognized as a significant cause of acute lower respiratory tract infections in young children, often resulting in hospitalizations [22]. In this study, we aimed to investigate the effect of autophagy inhibition on innate cytokine production in RSV-infected human lung epithelial cells. Specifically, we examined the effect of siRNA-mediated knockdown of the autophagy protein Bec-1, building upon our previous work that utilized pharmacological autophagy inhibitors [15].

Previous in vivo studies have implicated autophagy in the regulation of cytokine responses during RSV infections [10,11,12,13,14]. Studies using knockout mice lacking the Bec-1 protein showed that, following RSV infection, Bec-1 deficiency caused a reduction in the production of interferon (IFN)-β [12] and IFN-γ [11] in bone-marrow-derived dendritic cells (BMDCs) and pulmonary DCs, respectively. In a later year, the same research group reported that mice deficient in another autophagy-associated protein called microtubule associated protein 1 light chain 3 beta (map1-LC3b) also exhibited decreased RSV-induced IFN-γ production in whole lung lysates [14]. Similarly, another study revealed a decrease in IFN-β production when mouse macrophage RAW264.7 cells were transfected with Bec-1 siRNA [13]. Moreover, the inhibition of autophagy through pharmacological means, specifically using the autophagy inhibitor 3-methyladenine (3-MA), resulted in reduced production of cytokines IL-6 and CCL5 in BMDCs cultured from BL6 mice [10]. However, it is important to highlight that these studies were conducted in mouse models, underlying the need for investigations in human cells. To address this gap, we previously revealed for the first time that autophagy inhibition using pharmacological inhibitors did not affect the RSV-induced cytokine production in human lung epithelial cells [15].

In the present study, we aimed to validate our previous findings [15] using a more specific approach, that is, by knocking down the expression of the autophagy protein Bec-1 by siRNAs. Bec-1 is a key protein in autophagy signaling where it acts as a platform for the assembly of the phosphatidylinositol 3-kinase catalytic subunit type 3 (P13KC3) complex during the initiation stage of autophagosome formation [17,23]. Prior to assessing cytokine production, we first confirmed that the RNA interference approach successfully inhibited autophagy while leaving the cell viability undisturbed.

In our present study, we found that autophagy inhibition by siRNA-mediated Bec-1 knockdown did not affect the production of innate cytokines CXCL8 and CCL5 in RSV-infected human lung epithelial cells, thus validating our previous findings [15]. CCL5 is a chemoattractant cytokine that strongly attracts eosinophils, and eosinophilic inflammation is widely known to lead to airway obstruction in patients infected with RSV [21,24]. Our results regarding CCL5 production align with Reed et al. (2013), who demonstrated that Bec-1 deficiency in mice did not affect CCL5 production by murine lung epithelial cells [11]. In contrast, the study by Owczarczyk et al. (2015) showed that autophagy inhibition by 3-MA reduced CCL5 production in BMDCs cultured from BL6 mice [10]. Thus, it can be postulated that autophagy plays a role in regulating the innate cytokine production in immune cells such as dendritic cells but not in structural cells such as lung epithelial cells.

Apart from the fact that the previous in vivo studies measured cytokines produced by innate immune cells, specifically macrophages and dendritic cells [10,11,12,13,14], while our study measured cytokines produced by structural epithelial cells, another potential reason for the contradicting findings in the present study regarding the role of autophagy in cytokine production is that all the mouse studies utilized RSV antigenic subgroup A, whereas our study used RSV subgroup B.

In this study, we also measured CXCL8 production, an innate cytokine that none of the previously published mouse studies had assessed. CXCL8 is a potent chemokine for neutrophils, and neutrophilic inflammation is a prominent contributor of acute airway inflammation caused by RSV infection [21]. Consistent with the results obtained using pharmacological inhibitors [15], the inhibition of autophagy by siRNA knockdown of Bec-1 did not affect CXCL8 production in human lung epithelial cells.

However, our study has some limitations. Specifically, we only targeted one autophagy-associated protein using siRNA. Targeting other autophagy proteins such as map1-LC3b may further confirm our findings that autophagy inhibition did not affect RSV-induced cytokine production in human lung epithelial cells. Furthermore, investigating the effects of Bec-1 knockdown on the production of other innate cytokines, such as IFN-β, IFN-γ, CCL5 and IFN-6 as examined in previous publications, would provide a more comprehensive understanding of the role of autophagy in cytokine regulation during RSV infection. As mentioned earlier, it would be beneficial to support our data by utilizing RSV antigenic subgroup A.

## 5. Conclusions

Our findings demonstrate that autophagy inhibition via siRNA-mediated knockdown of autophagy protein Bec-1 does not affect RSV-induced innate cytokine production in human lung epithelial cells. This study provides further evidence that targeting autophagy may not be a suitable strategy to reduce RSV-induced airway inflammation.

## Data Availability

The data presented in this study are available on request from the corresponding author.
